# Trends in prevalence and disability-adjusted life years for refractive disorders in China and globally from 1990 to 2021: an analysis of the Global Burden of Disease Study 2021

**DOI:** 10.3389/fpubh.2025.1517056

**Published:** 2025-02-12

**Authors:** Fangfang Lai, Hongfang Xia, Liang Wang

**Affiliations:** ^1^College of Clinical and Medical Technology, Sichuan Vocational College of Health and Rehabilitation, Zigong, China; ^2^Department of Public Health, China University of Geosciences Wuhan Hospital, Wuhan, China; ^3^Department of Public Health, Wuhan Sports University Hospital, Wuhan, China

**Keywords:** refractive disorders, APC model, prevalence, disability-adjusted life years, GBD

## Abstract

**Objectives:**

This study aimed to describe the temporal trends in the prevalence and disability-adjusted life years (DALYs) of refractive disorders (RD) in China and globally from 1990 to 2021 and provide predictions in China for the next decade.

**Methods:**

Utilizing open data from the Global Burden of Disease database (2021), this study conducted a comprehensive comparative analysis of the RD burden in China and globally, including changes in prevalence and DALYs. Joinpoint regression was employed to calculate the annual percentage change, the average annual percentage change, and its corresponding 95% confidence interval to reflect segmented trends in RD burden. The APC model was used to assess the net effects of age, period, and cohort. The ARIMA model was applied to predict future trends.

**Results:**

The temporal trend of the health burden of RD in China aligned with the global trend, though the magnitude of change was greater, and the gap between the two had been narrowing recently. In China, the age-standardized DALYs rate for the total population (per 100,000 population) decreased from 74 in 1990 to 66 in 2021 and was projected to fall to 29.43 by 2031. The age-standardized prevalence rate for the total population (per 100,000 population) was expected to decrease to 411.23 by 2031, down from 1568 in 1990 to 1468 in 2021. However, the number of cases and the crude rates of prevalence and DALYs exhibited upward trends. In terms of age-specific rates, older adults exhibited higher prevalence and DALYs rates than younger adults. Regarding gender stratification, females had higher prevalence and DALYs rates than males.

**Conclusion:**

The age-standardized rates of prevalence and DALYs for RD have decreased in recent years and may continue to decline over the next decade. However, the crude rates of prevalence and DALYs are on the rise. The health burden of RD remains substantial, with females and the older population bearing a higher burden than males and younger populations.

## 1 Introduction

Refractive disorders (RD), including myopia, hyperopia, astigmatism, anisometropia, presbyopia, and aphakia, occur when the eye's ability to focus light on the retina is impaired, resulting in blurred vision. It is estimated that ~7.6 billion people worldwide are affected by RD, including 2.7 billion with uncorrected RD, 2.0 billion with corrected RD, and 2.9 billion without the need for vision correction (1). In China, the prevalence of RD is similarly concerning. The National Health Commission's report on the “14th 5-Year Plan for National Eye Health (2021–2025)” highlights the significant health burden of RD, particularly myopia, among children and adolescents (2). Uncorrected refractive errors are a major contributor to vision impairment both in China and globally (3, 4).

The economic impact of RD is considerable, as individuals often incur substantial costs for corrective lenses, eye care services, and potential lost wages due to vision impairment. Moreover, RD have been associated with an increased risk of ocular complications, such as macular degeneration, glaucoma, cataracts, and retinal detachment. RD also affect academic performance, work productivity, and overall quality of life (5–7). Older adults with vision impairment are at greater risk for adverse outcomes, including cognitive decline, dementia, depression, and falls (8–11). However, RD is a health issue that can be corrected or prevented, suggesting that early prevention and intervention can significantly reduce its disease burden (12).

The current prevalence and disease burden of RD represent a significant public health concern, affecting millions of individuals globally. Approximately 90% of individuals with uncorrected RD reside in developing countries. In 2018, China ranked second among the top 10 countries with the highest burden of uncorrected RD and is projected to rank third by 2025 (1). The prevalence of RD varies across regions, with particularly high rates in metropolitan areas, where lifestyle factors such as increased use of electronic devices and reduced outdoor activities have contributed to the rising prevalence of RD (13).

In response to these challenges, it is crucial to implement effective public health strategies that focus on increasing awareness, improving access to eye care services, and promoting regular eye examinations. Early detection and timely correction of RD can significantly improve the quality of life for affected individuals and substantially reduce the associated disease burden. Addressing this issue is vital for healthcare systems worldwide, given the increasing prevalence of refractive conditions. Despite the considerable public health implications of RD, several research gaps remain. There is a lack of comprehensive research on the temporal trend of RD, particularly concerning the net effects of age, period, and cohort. Furthermore, the future trajectory of the disease burden of RD remains uncertain. These gaps limit our ability to accurately identify the target population and design effective interventions.

The aim of this study was to address the existing gaps in understanding the health burden of RD in China. Specifically, we sought to estimate temporal trends in the prevalence and disability-adjusted life years (DALYs) associated with RD in China, stratified by age and gender, and to compare these trends with global data. By identifying high-risk groups and providing a comprehensive analysis of RD prevalence, we can develop targeted prevention strategies. This approach will help reduce disparities in the global burden of RD and facilitate more effective coverage for refractive errors (14, 15).

## 2 Methods

### 2.1 Data source

The data were extracted from the GBD 2021 dataset, a publicly accessible and comprehensive database that records disease burden indicators for 371 diseases and injuries, and 88 risk factors. This dataset spans a range of conditions, from infectious diseases to non-communicable conditions such as cardiovascular disease, cancer, and mental health disorders, covering 204 countries and territories and 811 sub-national locations from 1990 to 2021. The data sources, statistical models, and core results of GBD 2021 had been previously described in detail (16–18).

In this study, the Global Health Data Exchange (GHDx) GBD Results Tool was used to collect data on RD, including prevalence numbers, DALYs numbers, age-standardized prevalence rates (ASPR), age standardized DALYs rates (ASDALYR), crude prevalence rates, crude DALYs rates, and their corresponding 95% uncertainty interval (UI) for China and globally from 1990 to 2021. The GBD Tool was available at https://vizhub.healthdata.org/gbd-results/.

Given that the data used in this study are openly accessible, ethics approval and informed consent were not required.

### 2.2 Trend analysis of disease burden

This study conducted descriptive analyses and visualizations of the prevalence and DALYs trends of RD in China and globally, with a focus on gender and age differences. To more precisely reveal segmented trends in prevalence and DALYs of RD from 1990 to 2021, the Joinpoint 5.1.0.0 software, available on the National Cancer Institute's website (https://surveillance.cancer.gov/joinpoint/download, accessed on April 10, 2024), was employed for segmented regression analysis (19). This method can identify significant joinpoints, segmenting the overall long-term trends into distinct subsegments (20).

For each subsegment, the annual percentage change (APC) was calculated with 95% confidence intervals (CI), and the significance of each trend was assessed. Additionally, the average annual percentage change (AAPC) was calculated to summarize the overall trends. An uptrend in a subsegment was indicated if the lower boundary of the APC/AAPC 95% CI was >0; conversely, a downtrend was demonstrated if the upper boundary of the 95% CI was below 0.

The statistical analyses and visualizations of the data were performed using R statistical software (version 4.2.1) and Joinpoint software (version 4.9.1.0). A *P*<*0.05* was considered statistically significant.

### 2.3 Age-period-cohort model

The age-period-cohort (APC) model was utilized as a robust methodological framework to explain the temporal trends in prevalence and DALYs of RD from 1990 to 2021. Based on the APC model, the net trends of prevalence and DALYs were analyzed by examining the effects of age, period, and cohort (21, 22). To meet the requirements of APC analysis, the data were grouped into successive 5-year age groups (i.e., 0–4, 5–9, …, 95–99), consecutive 5-year periods from 1992 to 2021 (i.e., 1992–1996, 1997–2001, …, 2017–2021), and corresponding consecutive 5-year birth cohorts (i.e.,1897–1901, 1902–1906, …, 2017–2021). We then computed the net drift, local drift, longitudinal age curve, and relative risk (RR) for age, period, and cohort respectively. For APC analysis, the National Cancer Institute's online APC Web Tool, available at https://analysistools.cancer.gov/apc/, was used.

### 2.4 Correlation analysis with SDI

This study employed Pearson correlation to assess the strength and direction of linear relationships between the Sociodemographic Index (SDI) and disease burden indicators. SDI is a composite measure that reflects the development status of a country or region, calculated based on variables such as per capita income, average educational attainment, and fertility rates. A higher SDI value indicates a more advanced development status for a country or region, with values ranging from 0 to 1.

### 2.5 Autoregressive integrated moving average model

The autoregressive integrated moving average (ARIMA) model is a powerful statistical tool for time series analysis and forecasting. It combines two key components: the autoregressive (AR) model, which captures the relationship between an observation and a number of lagged observations, and the moving average (MA) model, which models the relationship between an observation and a residual error from a moving average model applied to lagged observations.

The ARIMA model serves as a robust framework for understanding and forecasting time-dependent data across various fields, including epidemiology, economics, and environmental studies. In previous GBD studies, ARIMA model had been widely used (23–25). Similarly, in this study, the ARIMA model was employed to forecast the prevalence and DALYs of RD in China for the next decade, providing insights into future trends in the health burden of RD.

## 3 Results

### 3.1 Temporal trends of RD prevalence and DALYs in China and globally

#### 3.1.1 Changes in prevalence between 1990 and 2021

**Figure 1:**
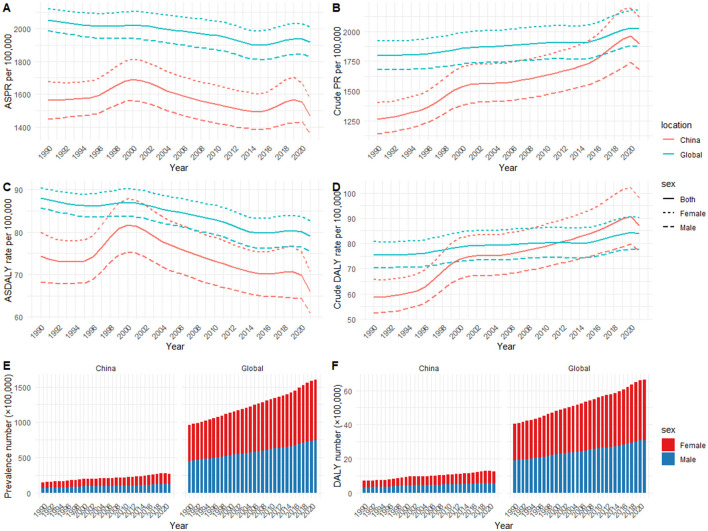
Temporal trends of health burden of refractive disorders in China and globally (1990–2021): **(A)** age-standardized prevalence rate; **(B)** crude prevalence rate; **(C)** age-standardized DALYs rate; **(D)** crude DALYs rate; **(E)** prevalence number; **(F)** DALYs number.

A comparison of the disease burden of RD in China and globally between 1990 and 2021 was presented in Figure 1. From 1990 to 2021, both the crude PR and the number of RD cases exhibited an overall upward trend in China and globally, with the China increase being noticeably larger (Figures 1B, E). Conversely, the ASPR of RD fluctuated between 1990 and 2021 in China and globally, with the global ASPR being higher than in China (Figure 1A).

Specifically, the number of RD cases in both sexes in china increased from 14,885,107 (95% UI: 13,287,105 to 16,601,891) in 1990 to 26,970,569 (95% UI: 23,579,348 to 30,840,249) in 2021; the crude PR per 100,000 people in both sexes increased from 1,265 (95% UI:1,129 to 1,411) in 1990 to 1,896 (95% UI:1,657 to 2,168) in 2021, with an overall AAPC of 1.35 (95% CI: 1.29 to 1.42); However, the ASPR per 100,000 people decreased from 1,568 (95% UI: 1,397 to 1,743) in 1990 to 1,468 (95% UI: 1,301 to 1,641) in 2021, with an overall AAPC of −0.18 (95% CI: −0.22 to −0.14; Table 1 and Supplementary Table 1).

**Table 1:** Prevalence in China and globally, 1990 and 2021.

**Characteristic**	**Number (95% UI)**		**Crude rate per 100,000 (95% UI)**		**Age-standardized rate per 100,000 (95% UI)**	
	**1990**	**2021**	**1990**	**2021**	**1990**	**2021**
**China**						
Male	6,905,722 (6,151,564–7,690,871)	12,230,869 (10,665,113–14,001,530)	1,138 (1,014–1,267)	1,680 (1,465–1,923)	1,449 (1,289–1,614)	1,357 (1,202–1,517)
Female	7,979,386 (7,127,875–8,888,313)	14,739,700 (12,926,426–16,845,104)	1,401 (1,251–1,560)	2,122 (1,861–2,425)	1,679 (1,494–1,867)	1,576 (1,398–1,762)
Both	14,885,107 (13,287,105–16,601,891)	26,970,569 (23,579,348–30,840,249)	1,265 (1,129–1,411)	1,896 (1,657–2,168)	1,568 (1,397–1,743)	1,468 (1,301–1,641)
**Global**						
Male	45,149,517 (40,528,116–49,979,248)	74,223,582 (66,109,341–83,068,548)	1,681 (1,509–1,861)	1,875 (1,670–2,098)	1,989 (1,780–2,200)	1,828 (1,632–2,033)
Female	50,828,803 (45,686,966–56,154,379)	85,542,335 (76,384,917–95,629,800)	1,920 (1,725–2,121)	2,176 (1,943–2,432)	2,123 (1,899–2,360)	2,010 (1,795–2,237)
Both	95,978,319 (86,236,197–106,044,403)	159,765,917 (142,526,915–178,698,348)	1,799 (1,617–1,988)	2,025 (1,806–2,264)	2,054 (1,835–2,276)	1,920 (1,715–2,135)

UI, uncertainty interval.

Globally, the prevalence in both sexes increased from 95,978,319 (95% UI: 86,236,197 to 106,044,403) cases in 1990 to 159,765,917 (95% UI: 142,526,915 to 178,698,348) cases in 2021; the crude PR per 100,000 people in both sexes increased from 1,799 (95% UI:1,617 to 1,988) in 1990 to 2,025 (95% UI:1,806 to 2,264) in 2021, with an overall AAPC of 0.39 (95% CI: 0.36 to 0.42); However, the ASPR in both sexes decreased from 2,054 (95% UI:1,835 to 2,276) per 100,000 people in 1990 to 1,920 (95% UI:1,715 to 2,135) per 100,000 people in 2021, with an overall AAPC of −0.21 (95% CI: −0.23 to −0.19; Table 1 and Supplementary Table 1).

#### 3.1.2 Changes in DALYs between 1990 and 2021

The number and the crude rate of DALYs due to RD in China and worldwide gradually increased from 1990 to 2021, with a significantly greater increase observed in China (Figures 1D, F). In contrast, the ASDALYR of RD in China and globally fluctuated and declined from 1990 to 2021, with the global ASDALYR being higher than in China (Figure 1C).

Specifically, the number of DALYs due to RD in both sexes in china increased from 692,062 (95% UI:495,982 to 958,459) in 1990 to 1,238,862 (95% UI:876,580 to 1,720,627) in 2021; the crude rate per 100,000 people in both sexes increased from 59 (95% UI:42 to 81) in 1990 to 87 (95% UI:62 to 121) in 2021, with an overall AAPC of 1.32 (95% CI:1.24 to 1.41); However, the age-standardized rate per 100,000 people in both sexes decreased from 74 (95% UI:54 to 102) in 1990 to 66 (95% UI:47 to 92) in 2021, with an overall AAPC of −0.34 (95% CI:-0.41 to −0.28; Table 2 and Supplementary Table 1).

**Table 2:** DALYs in China and globally, 1990 and 2021.

**Characteristic**	**Number (95% UI)**		**Crude rate per 100,000 (95% UI)**		**Age-standardized rate per 100,000 (95% UI)**	
	**1990**	**2021**	**1990**	**2021**	**1990**	**2021**
**China**						
Male	317,917 (225,630–444,007)	559,100 (393,922–777,820)	52 (37–73)	77 (54–107)	68 (49–94)	61 (43–85)
Female	374,144 (269,571–517,092)	679,761 (483,094–945,883)	66 (47–91)	98 (70–136)	80 (58–109)	71 (50–99)
Both	692,062 (495,982–958,459)	1,238,862 (876,580–1,720,627)	59 (42–81)	87 (62–121)	74 (54–102)	66 (47–92)
**Global**						
Male	1,893,306 (1,319,889–2,748,928)	3,069,277 (2,123,253–4,439,332)	70 (49–102)	78 (54–112)	86 (61–122)	76 (52–109)
Female	2,135,778 (1,498,780–3,063,246)	3,549,323 (2,475,790–5,089,344)	81 (57–116)	90 (63–129)	91 (64–128)	83 (58–119)
Both	4,029,084 (2,821,217–5,812,174)	6,618,600 (4,599,082–9,528,676)	76 (53–109)	84 (58–121)	88 (62–125)	79 (55–114)

DALYs, disability adjusted life years; UI, uncertainty interval.

Globally, the DALYs in both sexes increased from 4,029,084 (95% UI:2,821,217 to 5,812,174) in 1990 to 6,618,600 (95% UI:4,599,082 to 9,528,676) in 2021; the crude DALYs rate per 100,000 people in both sexes increased from 76 (95% UI:53 to 109) in 1990 to 84 (95% UI:58 to 121) in 2021, with an overall AAPC of 0.35 (95% CI:0.32 to 0.38); However, the ASDALYR in both sexes decreased from 88 (95% UI:62 to 125) per 100,000 people in 1990 to 79 (95% UI:55 to 114) per 100,000 people in 2021, with an overall AAPC of −0.33 (95% CI: −0.36 to −0.31; Table 2 and Supplementary Table 1).

### 3.2 Health burden of RD in different age groups in 1990 and 2021

**Figure 2:**
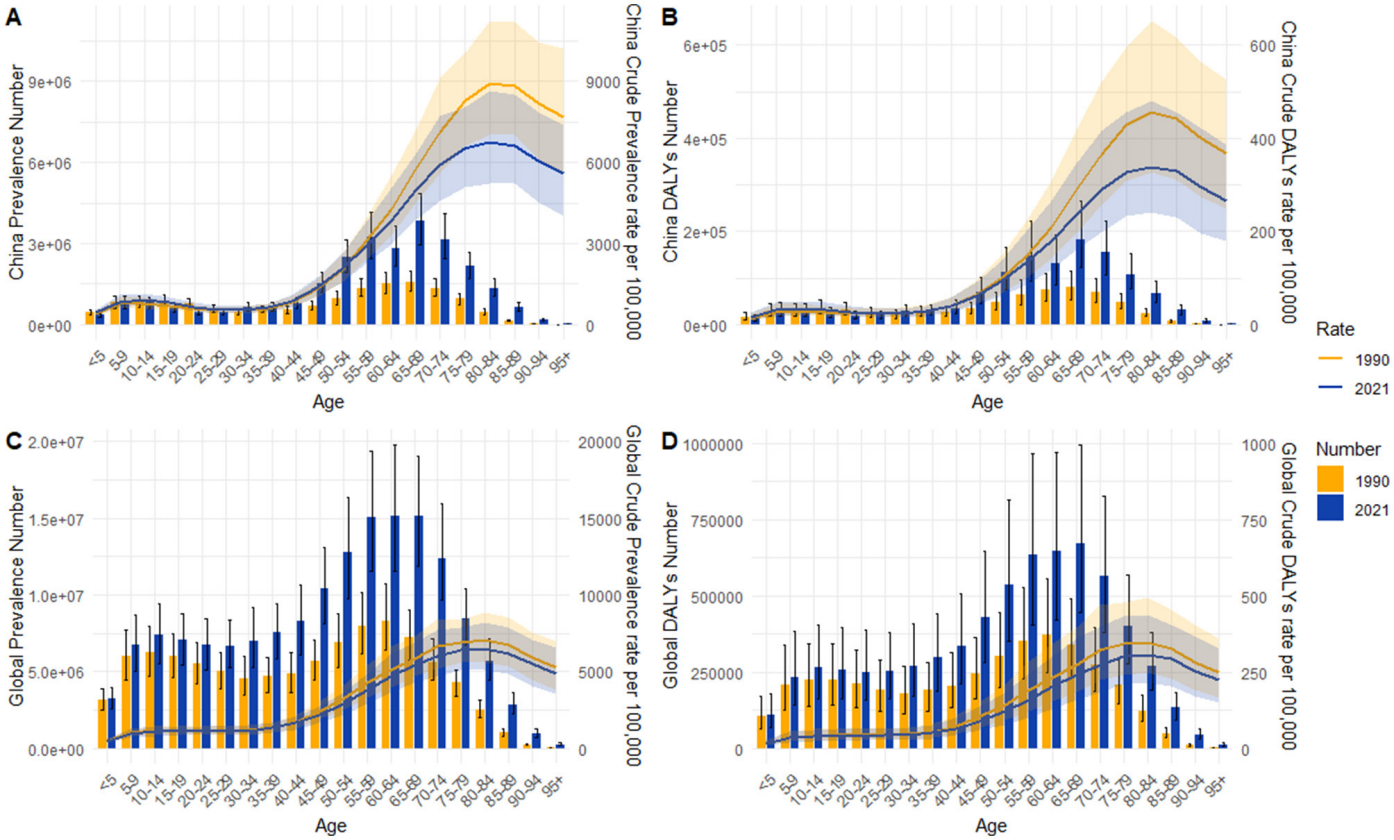
Age-specific rate and number for prevalence and DALYs of refractive disorders between 1990 and 2021: **(A)** prevalence in China; **(B)** DALYs in China; **(C)** global prevalence; **(D)** global DALYs.

Figure 2 presents a comparison of the prevalence cases and DALYs numbers of RD across different age groups in 1990 and 2021, along with their corresponding age-specific rates.

In terms of the prevalence, the highest number of cases in China was observed in the 65–69 age group, and the highest rate was in the 80–84 age group in both 1990 and 2021 (Figure 2A). The prevalence of RD among the older adult was notably higher than that among younger individuals. The global prevalence of RD exhibited a similar trend, with the highest number of cases in the 60–64 age group and highest age-specific prevalence rate in the 75–79 age group (Figure 2C).

Similar trends were observed in DALYs. In China, the age group with the highest number of DALYs was 65–69 in both 1990 and 2021. However, the peak DALYs rate occurred in the 80–84 age group in both years (Figure 2B). Globally, the age group with the highest number of DALYs was 60–64 in 1990, and 65–69 in 2021. The peak DALYs rate, however, occurred in the 75–79 age group in both 1990 and 2021(Figure 2D).

It was also worth noting that a small peak in RD burden was also observed in the 10–14 age group, both in China and globally.

### 3.3 Gender disparities in the health burden of RD

**Figure 3:**
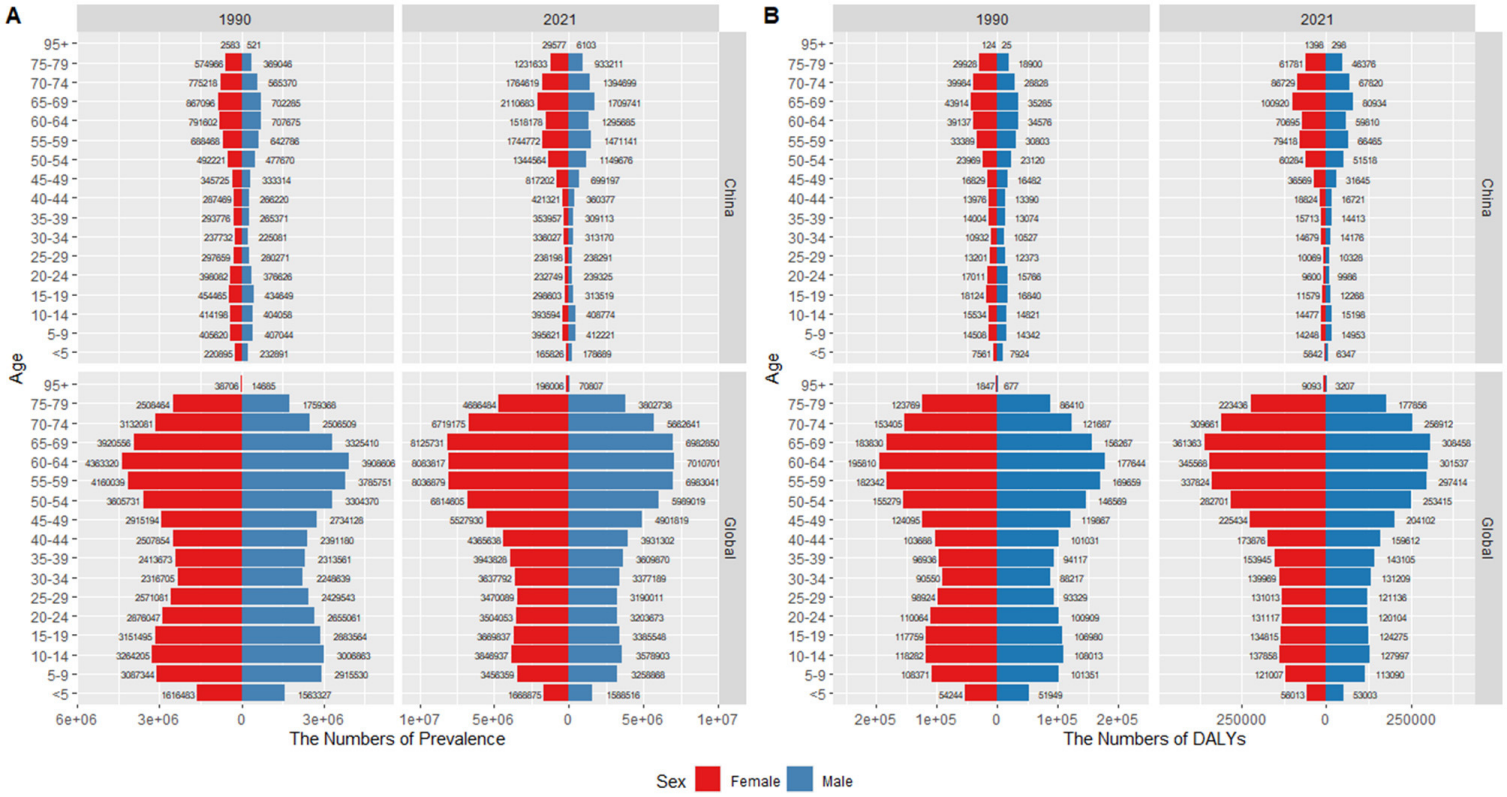
Age-sex pyramid distribution of refractive disorders in China and globally: **(A)** prevalence; **(B)** DALYs.

Figure 3 illustrates the prevalence cases and DALYs numbers of RD across different age groups by gender in 1990 and 2021. Notably, the distribution pattern of the prevalence and DALYs numbers among males across ages were consistent with that of females, while the number of females with RD exceeded that of males in most age groups. The peak number of prevalence and DALYs in males and females occurred in the same or similar age groups. As a result, the peak age was basically consistent with what was observed in the total population presented in Figure 2.

**Figure 4:**
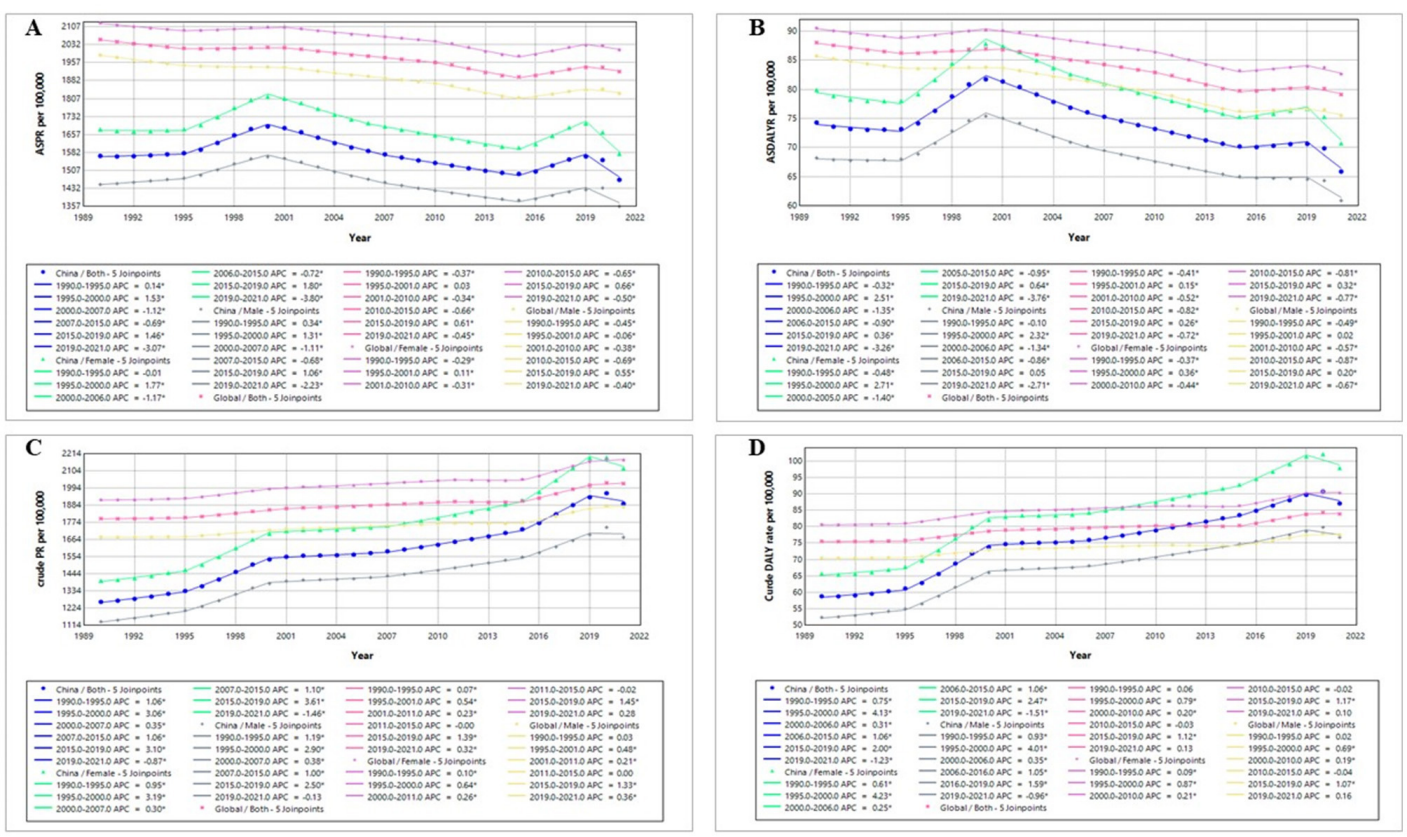
Joinpoint analysis of the health burden of refractive disorders in China and globally (1990–2021): **(A)** age-standardized prevalence rate; **(B)** age-standardized DALYs rate; **(C)** crude prevalence rate; **(D)** crude DALYs rate. Dots = annual rates; straight continuous line = statistical trend according to Joinpoint analysis; *indicates that the annual percent change (APC) is significantly different from zero at the alpha = 0.05 level.

Additionally, although the temporal trends for males and females were generally consistent with the total population, the rates of prevalence and DALYs for females were higher than those for males (Figures 1, 4).

### 3.4 Joinpoint regression analysis of the burden of RD in China and globally

The segmental trends of prevalence and DALYs rate of RD in China and globally from 1990 to 2021 were analyzed using joinpoint regression analysis (Figure 4).

The ASPR of RD in China exhibited a fluctuating declines for both sexes, with three major declines occurring during 2000–2007, 2007–2015, and 2019–2021, respectively (APC_2000 − 2007_ = −1.12, APC_2007 − 2015_ = −0.69, and APC_2019 − 2021_ = −3.07). In comparison, the global decline in ASPR of RD was more gradual, with four gentle descents during 1990–1995, 2001–2010, 2010–2015, and 2019–2021 (APC_1990 − 1995_ = −0.37, APC_2001 − 2010_ = −0.34, APC_2010 − 2015_ = −0.66, and APC_2019 − 2021_ = −0.45; Figure 4A). In contrast, the crude prevalence rate significantly increased since 1990, with the highest APCs observed in 2015–2019 in China (APC_2015 − 2019_ = 3.10) and globally (APC_2015 − 2019_ = 1.39; Figure 4C).

The similar trends were observed in the DALYs rates from 1990 to 2021. The ASDALYR of RD in China and globally displayed a fluctuating downward trend, with four major declines (China: APC_1990 − 1995_ = −0.32, APC_2000 − 2006_ = −1.35, APC_2006 − 2015_ = −0.90, and APC_2019 − 2021_ = −3.26; Global: APC_1990 − 1995_ = −0.41, APC_2001 − 2010_ = −0.52, APC_2010 − 2015_ = −0.82, and APC_2019 − 2021_ = −0.72; Figure 4B). Conversely, both the China and global crude DALYs rate showed an overall upward trend from 1990 to 2021, with the highest APCs in 1995–2000 in China (APC_1995 − 2000_ = 4.13) and in 2015–2019 globally (APC_2015 − 2019_ = 1.12; Figure 4D).

### 3.5 Age-period-cohort analysis on the burden of RD in China

**Figure 5:**
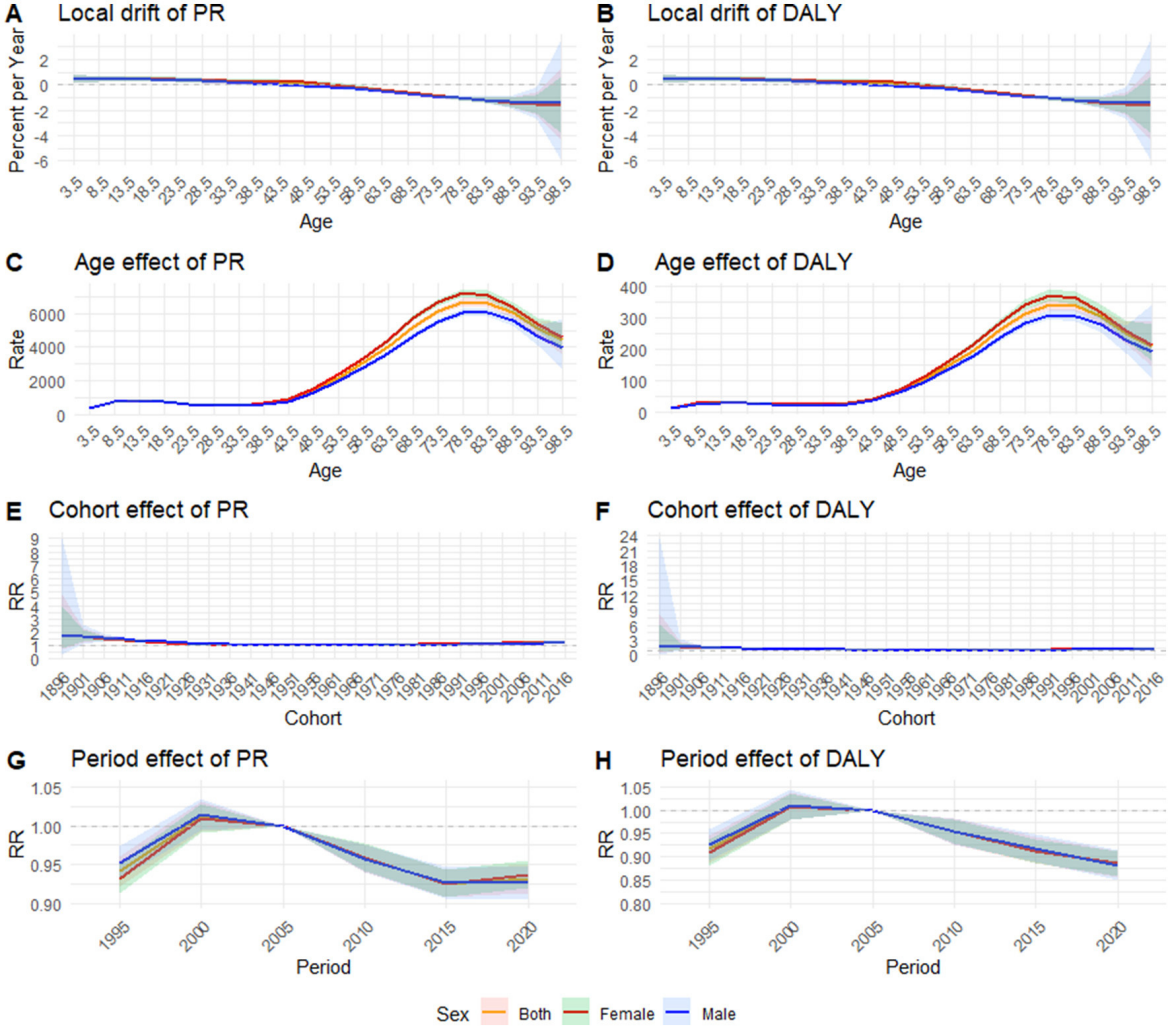
Age-period-cohort model for age standardized rate of prevalence and DALYs in China from 1992 to 2021: **(A)** local drift of prevalence; **(B)** local drift of DALYs; **(C)** age effect of prevalence; **(D)** age effect of DALYs; **(E)** cohort effect of prevalence; **(F)** cohort effect of DALYs; **(G)** period effect of prevalence; **(H)** period effect of DALYs; RR, relative risk.

We employed APC model to calculate the age-period-cohort effects of RD prevalence and DALYs rate in China, including net drifts, local drifts, age-based rates, cohort relative risks (RRs), and period RRs (Figure 5 and Supplementary Tables 2–6).

The net drift values represent the overall estimated annual percentage change. From 1990 to 2021, the net drifts for RD per year in China were −0.21% (95% CI: −0.30%, −0.11%) for the both sexes, −0.16% (95% CI: −0.24%, −0.07%) for females, and −0.26% (95% CI: −0.38%, −0.14%) for males. For DALYs, the net drifts per year in China were −0.30% (95% CI: −0.44%, −0.15%) for the both sexes, −0.27% (95% CI: −0.40%, −0.14%) for females, and −0.33% (95% CI: −0.52%, −0.13%) for males (Supplementary Table 2).

Local drift values represent the estimated annual percentage change for each age group. Unlike the net drift, which is a constant < 0, the local drift takes values within a certain range and decreases with age. As shown in Figure 5A, the local drifts of RD prevalence ranged from 0.48% (95% CI: 0.13%, 0.83%) in the 0–4 age group to −1.54% (95% CI: −4.28%, 1.27%) in the 95–99 age group for the both sexes, dropping below 0 after the 45–49 age group. After stratification by gender, the females local drifts ranged from 0.49% (95% CI: 0.28%, 0.69%) in the 10–14 age group to −1.61% (95% CI: −3.81%, 0.64%) in the 95–99 age group, dropping below 0 after the 50–54 age group; however, the males local drifts ranged from 0.48% (95% CI: 0.15%, 0.82%) in the 0–4 age group to −1.39% (95% CI: −2.63%, −0.14%) in the 90–94 age group, dropping below 0 after the 40–44 age group. As shown in Figure 5B, the local drifts of DALYs ranged from 0.72% (95% CI: 0.08%, 1.36%) in the 0–4 age group to −1.51% (95% CI: −5.77%, 2.93%) in the 95–99 age group for the both sexes, dropping below 0 after the 35–39 age group. After stratification by gender, the females local drifts ranged from 0.69% (95% CI: 0.04%, 1.35%) in the 0–4 age group to −1.58% (95% CI: −4.95%, 1.91%) in the 95–99 age group, dropping below 0 after the 40–44 age group; however, the males local drifts ranged from 0.74% (95% CI: 0.12%, 1.36%) in the 0–4 age group to −1.30% (95% CI: −8.44%, 6.40%) in the 95–99 age group, dropping below 0 after the 30–34 age group (Supplementary Table 3).

The longitudinal age curves of RD prevalence and DALYs rate by sex were shown in Figures 5C, D, respectively. In early ages (before 40–44), both prevalence and DALYs rates were lower. After age 40–44, the rates rapidly increased and reached their peak in the 75–79 age group for the total population [DALYs rate: 343.52 (95% CI: 326.96, 360.91) per 100,000 population; PR: 6674.34 (95% CI: 6460.67, 6895.09) per 100,000 population], then rapidly decreased. After stratification by gender, the peak rate of PR and DALYs remained in the 75–79 age group. The highest PR was 7212.18 (95%CI 6982.80–7449.09) per 100,000 population for the females and 6056.25 (95%CI 5857.68, 6261.54) per 100,000 population for the males. The highest DALYs rate was 372.15(95%CI 354.69, 390.48) per 100,000 population for the females and 310.44 (95%CI 294.87, 326.82) per 100,000 population for the males (Supplementary Table 4).

Figures 5E, F depicts the estimated cohort effects of RD in China. The overall change in cohort-based trends of RD prevalence and DALYs rate was relatively steady, having an ascent after a initial decline trend, with the lowest RR around the reference birth cohort. The early birth cohort had a greater RR, with the highest RR in the 1896–1901 birth cohort (DALYs rate for both sexes: RR_cohort1896 − 1901_ = 1.85, 95%CI 0.41–8.41; PR for both sexes: RR _cohort1896 − 1901_ = 1.82, 95%CI 0.69–4.79). After gender stratification, the trends in the males and the females were the same (Supplementary Table 5).

The estimated period effects were depicted in Figures 5G, H. The period-based trends of RD prevalence and DALYs rates first showed a significant increase, reaching its peak in period_1997 − 2002_ (DALYs rate for both sexes: RR_period1997 − 2002_ = 1.01, 95%CI 0.98–1.04; PR for both sexes: RR_period1997 − 2002_=1.01, 95%CI 0.99–1.03) and then slowly decreasing. Between 1990 and 2021, comparable period-based trends were observed in both the males and the females (Supplementary Table 6).

### 3.6 Correlations of ASRs with SDI

**Figure 6:**
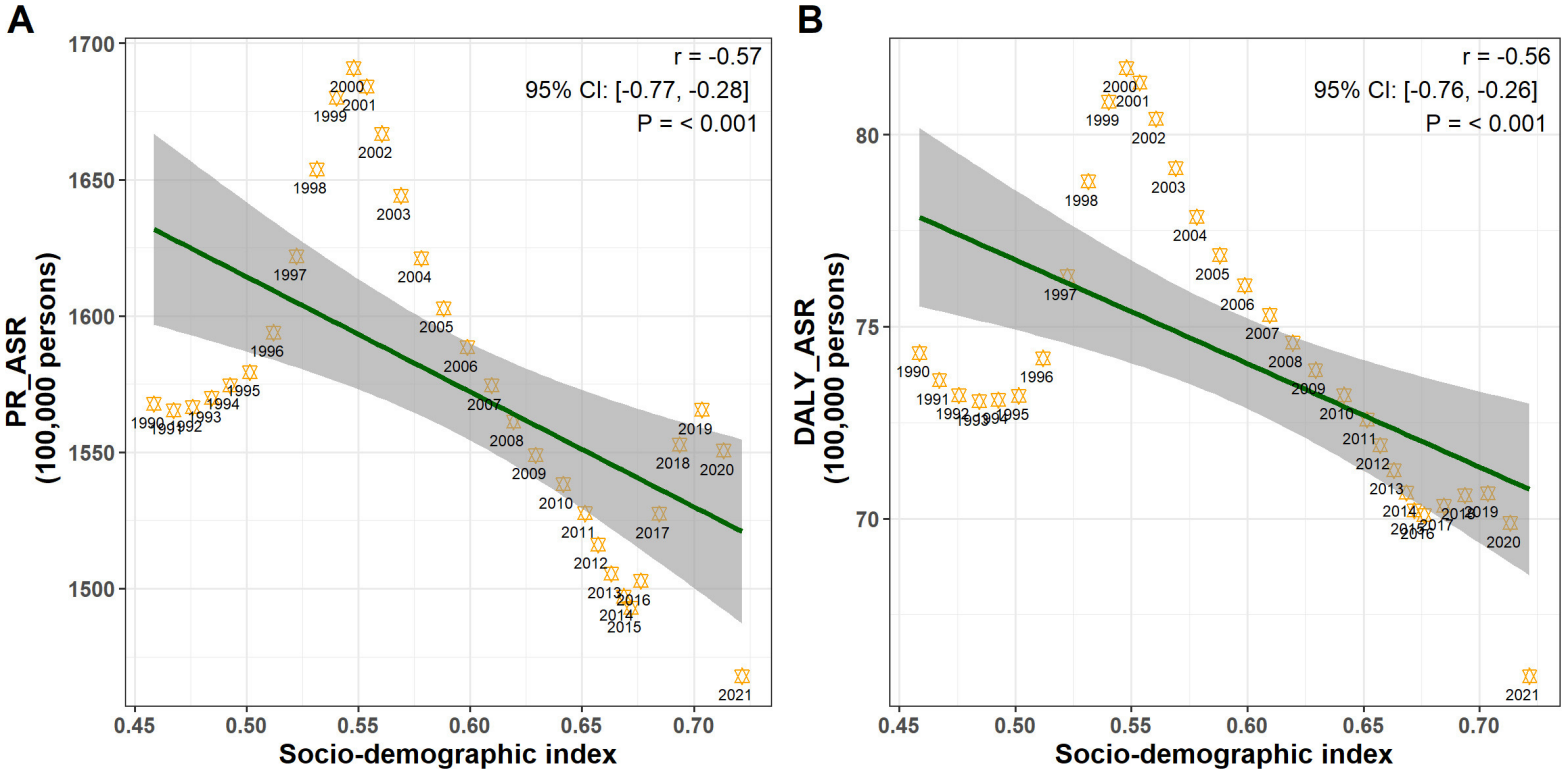
Pearson correlation analysis between SDI and age-standardized rates: **(A)** prevalence rate; **(B)** DALYs rate.

In this study, we examined the correlations between SDI values and age-standardized rates (ASRs), including ASPR and ASDALYR, from 1990 to 2021. Our analysis revealed a negative correlation between ASPR and SDI [*r* = −0.57 (95% CI: −0.77, −0.28), *P*<*0.001*], indicating that as SDI increases, ASPR decreases. Similarly, a negative correlation was observed between ASDALYR and SDI [*r* = −0.56 (95% CI: −0.76, −0.26), *P*<*0.001*], suggesting that improvements in sociodemographic factors are associated with a lower disease burden, as reflected by DALYs (Figure 6).

### 3.7 Prediction of the burden of RD in China in the next decade

The ARIMA model was used to quantitatively depict the trends of RD prevalence and DALYs over the following 10 years. In terms of the total population, after automatic filtering by the auto.arima() function, the optimized models were chosen to be the ARIMA model (2, 1, 1) for age-standardized PR with an AIC value of 230.73, and the ARIMA model (2, 1, 1) for age-standardized DALYs rate with an AIC value of 56.7. The optimized models were chosen to be the ARIMA model (2, 1, 1) for crude PR with an AIC value of 259.57, and the ARIMA model (1, 1, 1) for crude DALYs rate with an AIC value of 84.48. None of the residuals from above models shown statistical significance according to Ljung-Box test (model for age-standardized PR: χ^2^ = *7.528, P* = *0.995*; model for age-standardized DALYs rate: χ^2^ = *4.932, P* = *1.000*; model for crude PR: χ^2^ = *2.396, P* = *1.000*; model for crude DALYs rate: χ^2^ = *3.206, P* = *1.000*), indicating that the selected models fit well with the data.

**Figure 7:**
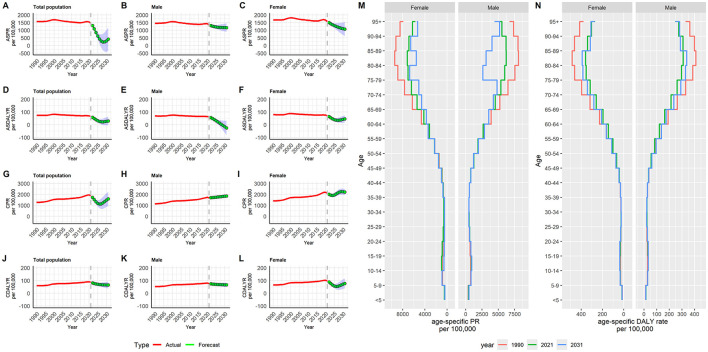
Predicted trends of refractive disorders prevalence and DALYs rate in China over the next 10 years (2022–2031): **(A)** ASPR of total population; **(B)** ASPR of male; **(C)** ASPR of female; **(D)** ASDALYR of total population; **(E)** ASDALYR of male; **(F)** ASDALYR of female; **(G)** CPR of total population; **(H)** CPR of male; **(I)** CPR of female; **(J)** CDALYR of total population; **(K)** CDALYR of male; **(L)** CDALYR of female; **(M)** age-specific prevalence rate; **(N)** age-specific DALYs rate. Red lines represent the true trends of refractive disorders prevalence and DALYs rate during 1990 to 2021; green dot lines and shaded regions represent the predicted trends and its 95%CI. ASPR, age-standardized prevalence rate; ASDALYR, age-standardized DALYs rate; CPR, crude prevalence rate; CDALYR, crude DALYs rate.

From 2022 to 2031, the ASPR and ASDALYR of RD showed downward trends in total population. The ASPR was expected to decrease from 1304.83 (95%CI: 1289.31, 1320.36) per 100,000 population in 2022 to 411.23 (95%CI: 0, 1181.05) per 100,000 population in 2031. The predicted ASDALYR also kept decline over the next decade, decreasing from 58.20 (95%CI: 57.23, 59.17) per 100,000 in 2022 to 29.43 (95%CI: 0, 63.29) per 100,000 in 2031. The crude prevalence rate was expected to slightly decrease from 1732.43 (95%CI: 1706.19, 1758.66) per 100,000 population in 2022 to 1589.92 (95%CI: 952.55, 2227.29) per 100,000 population in 2031. The predicted crude DALY rate decreases from 81.17 (95%CI: 79.50, 82.84) per 100,000 in 2022 to 64.69 (95%CI: 37.86, 91.53) per 100,000 in 2031. Similar trends over the next decade also were observed in the males and females (Figures 7A–L, Supplementary Tables 7, 8). From 2021 to 2031, it is expected that the changes in RD prevalence and DALYs will vary among different age groups. The change in disease burden among the older adult is much greater than that of young people. Until 2031, the older adult are still the main contributing factor to the disease burden (Figures 7M, N, Supplementary Tables 9, 10).

## 4 Discussion

This study investigated trends in the health burden of RD in China from 1990 to 2021 and compared them with global trends. To our knowledge, this is the first analysis of RD burden trends in China using Joinpoint regression analysis in conjunction with the APC model. Previous studies have primarily explored the global health burden of RD, utilizing data from the GBD 2017 or GBD 2019. These studies focused on evaluating the health burden of RD by factors such as year, age, region, gender, socioeconomic status, and other national characteristics, as well as analyzing its socioeconomic inequalities (15, 23). We also identified discrepancies in the data following the release of GBD 2021, which could alter the previous understanding of the health burden of RD. GBD 2019 reported the global ASDALYR for RD as 88.9 (95% UI: 60.5, 120.3) in 1990, while GBD 2021 reported the global ASDALYR for RD as 88.0 (95% UI: 62.0, 125.0) for the same year. This discrepancy may be attributed to improvements in the algorithms and models used in the analysis. Therefore, it is crucial to assess the health burden of RD in China using the most recent data from GBD 2021.

From 1990 to 2021, the proportion of RD cases in China increased from 15.5 to 16.9% globally. The proportion of DALYs in China increased from 17.2 to 18.7% globally. Among 61 countries and regions in Asia, China's DALYs crude rate ranking for RD has risen from 32nd to 13th, with China's DALYs number consistently ranking among the top 3 (i.e., second; Supplementary Figures 1, 2). Compared to most countries and regions in Asia, the burden of RD in China is becoming increasingly significant. Among the six subcategories of blindness and vision loss in China, both the DALYs crude rate and number have consistently ranked second (Supplementary Figure 3), indicating the need for heightened attention to RD within the broader context of blindness and vision loss. Although there are significant differences in the burden of RD among different countries and regions, a study conducted in China did not observe such differences (26), suggesting that geographical factors may not directly affect RD burden, but rather exert their influence through other potential factors such as race, education and economic level.

According to the current research, although the crude rate and the total number of cases exhibited an overall upward trend, there was an overall decline in the ASPR and ASDALYR for RD in China from 1990 to 2021. Similar patterns were observed in the global population. The decrease in age-standardized rates may be related to the continuous improvement in social development (e.g., SDI) and advancements in ophthalmic care, including early detection and management strategies, which may effectively reduce the prevalence and impact of RD among specific age groups. The increase in crude rates may be mainly attributed to changes in population structure, which result in a doubling of the number of patients in particular age groups, however, the standardized rate may not show significant changes (e.g., age group of 30–60 years in China). Research shows, over the past three decades, population aging has significantly altered the demographic structure (3). As the population grows and ages, the number of individuals affected by RD is likely to increase substantially (4). Furthermore, the prevalence and DALYs across all age groups increased significantly from 1990 to 2021. However, the decline in age-specific prevalence rates and DALYs rates was more pronounced among middle-aged and older individuals than among younger populations, further indicating that aging is a key factor influencing the health burden of RD. Although ASPR and ASDALYR for RD in China were lower than the global averages, the increase in the crude rate of PR and DALYs in China was considerably higher than the global rate, suggesting that RD burden in China may be disproportionately affected by population aging.

Notably, several significant turning points occurred between 1990 and 2021. The jointpoint regression analysis revealed that the ASPR and ASDALYR shifted abruptly from an upward to a downward trend after 2000, while the upward trend of the crude rate decelerated, potentially attributable to a decline in age-specific rates. Similarly, turning points were observed in 2015, where a downward trend reversed to an upward trend, and in 2019, where the trend reversed again from upward to downward. The former may be associated with the widespread use of electronic devices, while the latter may be linked to the COVID-19 pandemic. However, some studies suggest that home confinement during the pandemic affected people's lifestyles, leading to an increase in RD among children (27, 28). Further research is required to determine whether the impact of the COVID-19 pandemic varies across different age groups.

Our study also demonstrated that the health burden of RD exhibits two peaks across different age groups, with a smaller peak in the 5–9 age group and a larger peak in the 65–69 age group. This suggests the need for increased attention to both children and the older adult in future healthcare planning. Since RD primarily encompasses myopia, hyperopia, astigmatism, and presbyopia, the overall trends in the health burden of RD reflect these conditions collectively. Myopia is highly prevalent among young individuals and tends to stabilize between 15 and 16 years of age (29). In China, myopia is also the most common type of RD among school children (30, 31). Hyperopia is more common in younger children and typically decreases with age as the lens of the eye loses elasticity. Astigmatism often coexists with myopia or hyperopia. In young children, myopia and astigmatism remain the leading causes of uncorrected RD worldwide (32). Presbyopia, however, affects nearly everyone over the age of 40, with a global prevalence reaching ~85% in this age group (33, 34). With the aging of the population, the number of individuals affected by presbyopia is likely to increase further (35). Given that myopia cannot be completely cured, the health burden of RD in the older population is influenced by both myopia and presbyopia. Cataract and glaucoma surgeries may also contribute to the high prevalence of RD in the older adult (13, 36). The rise in myopia due to lifestyle and environmental changes, coupled with the increase in presbyopia due to population aging, are the primary drivers of the RD disease burden.

In terms of gender differences, females consistently display a greater RD burden than males. Similar findings have been reported in previous studies (37). Based on the GBD 2019, the overall prevalence of visual loss in women was also higher than that in men in China (4). Several factors contribute to the gender disparity in RD. Hormonal fluctuations during pregnancy have been implicated in the development and progression of RD (37). A genetic component also plays a significant role, with the heritability of refractive errors in females being higher than in males (38, 39). Gender-based disparities in healthcare access exacerbate this issue (40). Consequently, special attention should also be given to women.

The results of the APC model analysis indicated that, although the ASPR and ASDALYR exhibited an overall downward trend for the total population, significant differences remained across various age groups. Among the younger population, the standardization rates trended upward over time, whereas the middle-aged and older populations showed a downward trend. This may be associated with increased exposure of younger individuals to electronic devices. The study also revealed considerable variations in disease burden across different age groups, with the middle-aged and older populations bearing a significantly higher burden compared to younger groups. These findings suggest that the current disease burden of RD is primarily attributed to the middle-aged and older populations. However, the increasing health burden of RD among younger individuals should not be overlooked. Regarding the effects of age, period, and cohort, there was little variation in disease burden among different birth cohorts. The burden was more pronounced across different periods, showing an overall downward trend after 2005. This trend may be linked to the government's growing focus on RD and improvements in medical care in recent years.

Based on data from 1990 to 2021, this study predicts the future health burden of RD in China. Consistent with previous research findings (23), we also found that the prevalence and DALYs rates for RD are expected to continue declining over the next decade. However, as noted earlier, the actual health burden of RD should not be underestimated due to the increasing prevalence of myopia and an aging population. The prevention of RD should focus on high-risk populations and specific risk factors. According to our findings, the high-risk groups primarily include older women, older men, girls, and boys.

Previous studies have shown that RD are influenced by a variety of risk factors, including environmental, genetic, lifestyle, ocular health, and systemic health factors. Genetics plays a significant role in RD, with familial aggregation suggesting a genetic link (41). Insufficient outdoor activity increase the risk of myopia (42, 43). Certain ocular conditions, such as diabetic retinopathy, can affect refractive status and exacerbate the burden of RD (44). Systemic conditions, including hormonal imbalances (particularly during pregnancy), can also influence refractive development (45). Based on a comprehensive understanding of the risk factors and high-risk groups for RD, specific measures should be implemented in the future. Promoting healthy lifestyles among all high-risk population, such as encouraging outdoor activities, can help reduce the risk of RD. Regular eye examinations should be encouraged, especially for children, adolescents, and individuals with a family history of RD, to facilitate early detection and correction of RD. It is also important to monitor and manage systemic conditions that may affect refractive status and to educate individuals on maintaining overall health to protect their ocular health. Raising public awareness and educating the community about RD risk factors and preventive measures through campaigns and programs can further encourage individuals to seek professional advice and treatment when necessary. Additionally, this study identifies a negative correlation between China's SDI and both ASPR and ASDALYR from 1990 to 2021. This suggests that improving SDI levels may contribute to alleviating the overall disease burden of RD.

This study has several limitations. First, due to the reliance on existing GBD data for secondary analysis, there is a potential for misclassification bias. Second, during regular updates to the GBD database, new statistical methods and models may be introduced, which could result in discrepancies between data from previous and updated versions. Third, GBD 2021 does not provide separate disease burden data for specific refractive disorders such as myopia, hyperopia, astigmatism, anisometropia, presbyopia, and aphakia, thus limiting the ability to compare their prevalence and DALYs in China. As a result, only the overall health burden of RD could be analyzed. Fourth, GBD 2021 does not offer detailed data at the provincial or city level in China, preventing the analysis of regional differences in the health burden of RD and restricting the exploration of its spatial distribution. Finally, due to the limited demographic data available in the GBD database, it is not possible to fully examine the relationships between factors such as occupation, education, ethnicity, and disease burden. Furthermore, the GBD database lacks risk factor data for RD, which precludes the ability to conduct a risk factor analysis for these disorders.

Future research on RD should focus more on gender and age disparities through longitudinal studies that track trends, investigate genetic factors influencing gender differences, and examine the socioeconomic impacts on access to ophthalmic care. Investigating the effects of environmental factors, such as screen time and early childhood vision screening programs, as well as cultural variations, could offer valuable insights into the growing burden.

## 5 Conclusion

The findings indicate the age-standardized rates of PR and DALYs of RD have decreased in recent years and may continue to alleviate in the next decades. However, the crude rates of PR and DALYs are on the rise. The health burden of RD is still heavy, with females having a higher burden than males and the older population having a higher burden than young people. These findings suggest that special attention should be given to these populations, who may experience disproportionate impacts due to factors like gender differences and aging. Targeted public health interventions are also essential for reducing the overall burden and improving long-term health outcomes associated with RD.

## Data Availability

Publicly available datasets were analyzed in this study. This data can be found here: https://vizhub.healthdata.org/gbd-results/.
