# Supplementary material for: Trends in prevalence and disability-adjusted life years for refractive disorders in China and globally from 1990 to 2021: an analysis of the Global Burden of Disease Study 2021
Source: Front Public Health. 2025 Feb 12;13:1517056. doi: 10.3389/fpubh.2025.1517056 (PMC11860090; doi:10.3389/fpubh.2025.1517056)
Supplement: Supplementary file 1 [file Data_Sheet_1.pdf]

Supplementary Material

Supplementary Table 1. The AAPC for rate of prevalence and DALYs in China and globally from 1990 to 2021

Characteristics	Prevalence		DALYs	
	AAPC for Crude rate (95% CI)	AAPC for Age-standardized rate (95% CI)	AAPC for Crude rate (95% CI)	AAPC for Age-standardized rate (95% CI)
**China**				
Male	1.31 (1.26 , 1.37)	-0.17 (-0.21 , -0.14)	1.29 (1.23 , 1.35)	-0.33 (-0.38 , -0.28)
Female	1.38 (1.31 , 1.46)	-0.17 (-0.22 , -0.13)	1.35 (1.25 , 1.44)	-0.35 (-0.43 , -0.27)
Both	1.35 (1.29 , 1.42)	-0.18 (-0.22 , -0.14)	1.32 (1.24 , 1.41)	-0.34 (-0.41 , -0.28)
**Global**				
Male	0.36 (0.33 , 0.39)	-0.26 (-0.28 , -0.24)	0.32 (0.29 , 0.34)	-0.4 (-0.42 , -0.38)
Female	0.41 (0.37 , 0.45)	-0.17 (-0.19 , -0.15)	0.38 (0.34 , 0.41)	-0.28 (-0.31 , -0.26)
Both	0.39 (0.36 , 0.42)	-0.21 (-0.23 , -0.19)	0.35 (0.32 , 0.38)	-0.33 (-0.36 , -0.31)

DALYs: disability adjusted life years; AAPC: average annual percentage change; CI: confidence interval.

Supplementary Table 2. NET drift for prevelance and DALYs

Sex	Net Drift (%/year) for prevelance (95% CI)	Net Drift (%/year) for DALYs (95% CI)
Both	-0.21(-0.3, -0.11)	-0.3(-0.44, -0.15)
Female	-0.16(-0.24, -0.07)	-0.27(-0.4, -0.14)
Male	-0.26(-0.38, -0.14)	-0.33(-0.52, -0.13)

DALYs: disability adjusted life years; CI: confidence interval.

Supplementary Table 3. Local drift for prevelance and DALYs

Age (years)	Local Drift (%/year) for prevelance(95% CI)			Local Drift (%/year) for DALYs(95% CI)		
	**Both sexes**	**Female**	**Male**	**Both sexes**	**Female**	**Male**
0-4	0.48(0.13, 0.83)	0.48(0.12, 0.85)	0.48(0.15, 0.82)	0.72(0.08, 1.36)	0.69(0.04, 1.35)	0.74(0.12, 1.36)
5-9	0.45(0.22, 0.67)	0.47(0.24, 0.71)	0.43(0.22, 0.65)	0.59(0.18, 0.99)	0.58(0.16, 0.99)	0.61(0.21, 1.01)
10-14	0.45(0.26, 0.65)	0.49(0.28, 0.69)	0.44(0.25, 0.63)	0.5(0.15, 0.85)	0.5(0.14, 0.85)	0.53(0.18, 0.88)
15-19	0.42(0.23, 0.61)	0.45(0.25, 0.65)	0.41(0.23, 0.6)	0.39(0.05, 0.72)	0.37(0.03, 0.71)	0.43(0.09, 0.76)
20-24	0.4(0.21, 0.59)	0.41(0.22, 0.61)	0.39(0.21, 0.58)	0.3(-0.02, 0.62)	0.27(-0.06, 0.59)	0.34(0.02, 0.67)
25-29	0.35(0.16, 0.53)	0.37(0.18, 0.56)	0.33(0.14, 0.51)	0.22(-0.09, 0.52)	0.2(-0.11, 0.5)	0.24(-0.07, 0.55)
30-34	0.26(0.08, 0.44)	0.31(0.12, 0.49)	0.2(0.02, 0.39)	0.11(-0.18, 0.41)	0.13(-0.17, 0.42)	0.09(-0.21, 0.39)
35-39	0.21(0.04, 0.39)	0.28(0.11, 0.46)	0.11(-0.07, 0.29)	0.02(-0.26, 0.31)	0.08(-0.2, 0.36)	-0.05(-0.34, 0.23)
40-44	0.16(0.01, 0.32)	0.27(0.11, 0.43)	0.03(-0.13, 0.19)	-0.07(-0.32, 0.19)	0.03(-0.22, 0.28)	-0.18(-0.44, 0.08)
45-49	0.09(-0.04, 0.23)	0.21(0.07, 0.35)	-0.06(-0.2, 0.08)	-0.17(-0.39, 0.04)	-0.07(-0.28, 0.14)	-0.31(-0.53, -0.09)
50-54	-0.02(-0.14, 0.11)	0.08(-0.04, 0.21)	-0.16(-0.29, -0.04)	-0.29(-0.48, -0.09)	-0.2(-0.39, -0.01)	-0.41(-0.61, -0.21)
55-59	-0.2(-0.32, -0.09)	-0.13(-0.24, -0.01)	-0.32(-0.44, -0.2)	-0.42(-0.6, -0.24)	-0.37(-0.54, -0.19)	-0.52(-0.7, -0.34)
60-64	-0.43(-0.54, -0.32)	-0.37(-0.48, -0.26)	-0.51(-0.63, -0.4)	-0.57(-0.74, -0.4)	-0.53(-0.69, -0.36)	-0.64(-0.81, -0.46)
65-69	-0.64(-0.75, -0.53)	-0.6(-0.71, -0.49)	-0.69(-0.81, -0.58)	-0.7(-0.87, -0.53)	-0.67(-0.84, -0.51)	-0.74(-0.91, -0.57)
70-74	-0.85(-0.97, -0.72)	-0.81(-0.93, -0.69)	-0.87(-0.99, -0.74)	-0.83(-1.01, -0.64)	-0.8(-0.98, -0.63)	-0.83(-1.03, -0.64)
75-79	-1.04(-1.19, -0.89)	-1.01(-1.15, -0.87)	-1.04(-1.2, -0.87)	-0.95(-1.18, -0.73)	-0.93(-1.14, -0.72)	-0.93(-1.18, -0.68)
80-84	-1.2(-1.42, -0.99)	-1.18(-1.38, -0.98)	-1.19(-1.43, -0.94)	-1.07(-1.4, -0.75)	-1.05(-1.34, -0.76)	-1.04(-1.42, -0.66)
85-89	-1.37(-1.75, -0.99)	-1.37(-1.7, -1.04)	-1.33(-1.8, -0.86)	-1.24(-1.81, -0.66)	-1.24(-1.73, -0.74)	-1.19(-1.92, -0.45)
90-94	-1.5(-2.37, -0.62)	-1.53(-2.26, -0.8)	-1.39(-2.63, -0.14)	-1.4(-2.75, -0.04)	-1.43(-2.54, -0.31)	-1.3(-3.24, 0.67)
95-99	-1.54(-4.28, 1.27)	-1.61(-3.81, 0.64)	-1.32(-5.91, 3.51)	-1.51(-5.77, 2.93)	-1.58(-4.95, 1.91)	-1.3(-8.44, 6.4)

DALYs: disability adjusted life years; CI: confidence interval.

Supplementary Table 4. Age effect for prevelance and DALYs by APC model

Age (years)	prevelance rate (95% CI)			DALYs rate (95% CI)		
	**Both sexes**	**Female**	**Male**	**Both sexes**	**Female**	**Male**
0-4	347.11(324.51, 371.29)	352.7(328.92, 378.2)	344.41(322.45, 367.86)	12.46(11.08, 14.01)	12.84(11.39, 14.47)	12.17(10.83, 13.67)
5-9	720(681.42, 760.75)	740.2(699.64, 783.12)	706.63(669.13, 746.23)	26.75(24.37, 29.36)	27.91(25.41, 30.66)	25.82(23.52, 28.35)
10-14	819.3(778.56, 862.18)	849.64(806.69, 894.89)	796.69(757.21, 838.24)	31.02(28.48, 33.78)	32.61(29.94, 35.51)	29.69(27.24, 32.36)
15-19	743.12(707.68, 780.34)	772.4(735.09, 811.6)	719.63(685.29, 755.68)	29.31(27.03, 31.78)	30.81(28.43, 33.4)	28(25.8, 30.4)
20-24	592.75(565.43, 621.39)	613.86(585.23, 643.88)	575.4(548.84, 603.25)	24.93(23.08, 26.94)	26.05(24.13, 28.13)	23.95(22.14, 25.9)
25-29	512.95(490.48, 536.45)	535.06(511.34, 559.87)	494.31(472.63, 516.99)	22.58(21, 24.28)	23.6(21.96, 25.36)	21.69(20.15, 23.35)
30-34	509.81(488.66, 531.87)	542.75(520.08, 566.42)	480.45(460.4, 501.38)	23.3(21.77, 24.93)	24.62(23.02, 26.34)	22.11(20.64, 23.69)
35-39	606.42(583.95, 629.76)	663.72(639.07, 689.33)	553.71(532.96, 575.28)	28.28(26.64, 30.02)	30.42(28.67, 32.27)	26.32(24.76, 27.97)
40-44	849.96(822.39, 878.45)	937.3(906.88, 968.74)	768.51(743.24, 794.64)	40.08(38.06, 42.21)	43.39(41.23, 45.67)	37.01(35.1, 39.02)
45-49	1428.65(1389.41, 1468.99)	1552.39(1509.51, 1596.49)	1312.62(1276.28, 1349.99)	67.65(64.77, 70.66)	72.69(69.63, 75.89)	62.92(60.2, 65.77)
50-54	2177.59(2123.88, 2232.67)	2362.42(2303.83, 2422.5)	2000.97(1951.2, 2052.01)	103.98(100.03, 108.09)	112.38(108.16, 116.76)	95.95(92.23, 99.83)
55-59	3073.2(3001.05, 3147.08)	3350.3(3271.42, 3431.09)	2803.99(2737.44, 2872.16)	148.5(143.16, 154.04)	161.88(156.15, 167.82)	135.5(130.5, 140.68)
60-64	4025.09(3931.07, 4121.35)	4402.87(4299.92, 4508.28)	3651.67(3565.23, 3740.2)	197.86(190.76, 205.21)	216.41(208.79, 224.3)	179.51(172.9, 186.38)
65-69	5207.41(5065.29, 5353.51)	5713.76(5557.53, 5874.38)	4691.65(4562, 4824.98)	260.68(249.86, 271.97)	285.33(273.69, 297.46)	235.51(225.47, 246)
70-74	6127.24(5948.17, 6311.7)	6684.39(6488.96, 6885.69)	5533.65(5369.56, 5702.76)	312.71(298.88, 327.17)	340.94(326.15, 356.4)	282.54(269.67, 296.02)
75-79	6674.34(6460.67, 6895.09)	7212.18(6982.8, 7449.09)	6056.25(5857.68, 6261.54)	343.52(326.96, 360.91)	372.15(354.69, 390.48)	310.44(294.87, 326.82)
80-84	6639.26(6391.46, 6896.65)	7100.57(6842.53, 7368.34)	6036.97(5799.16, 6284.53)	338.27(319.35, 358.32)	362.39(343.03, 382.85)	306.26(287.9, 325.8)
85-89	6052.6(5746.54, 6374.95)	6361.03(6061.27, 6675.61)	5570.08(5248.92, 5910.89)	302.39(279.55, 327.1)	317.45(295.52, 341.01)	277.89(253.62, 304.49)
90-94	5110.77(4658.09, 5607.45)	5343.3(4932.13, 5788.74)	4633.62(4087.2, 5253.1)	248.62(215.77, 286.47)	258.21(229.01, 291.12)	228.15(187.87, 277.05)
95-99	4391.96(3525.12, 5471.96)	4557.49(3815.6, 5443.62)	3929.28(2715.59, 5685.4)	208.03(147.86, 292.71)	214(163.23, 280.57)	191.02(107.2, 340.38)

DALYs: disability adjusted life years; CI: confidence interval; APC model: Age-period-cohort model.

Supplementary Table 5. Cohort effect of prevelance and DALYs

Cohort	Relative risk of prevelance (95% CI)			Relative risk of DALYs (95% CI)		
	Both sexes	Female	Male	Both sexes	Female	Male
1896-1901	1.82(0.69, 4.79)	1.82(0.84, 3.95)	1.75(0.34, 9.04)	1.85(0.41, 8.41)	1.86(0.56, 6.12)	1.76(0.13, 23.53)
1901-1906	1.7(1.26, 2.29)	1.69(1.32, 2.17)	1.68(1.1, 2.58)	1.71(1.08, 2.72)	1.71(1.17, 2.5)	1.69(0.86, 3.3)
1906-1911	1.57(1.39, 1.78)	1.55(1.39, 1.73)	1.58(1.35, 1.86)	1.58(1.3, 1.91)	1.56(1.32, 1.85)	1.58(1.24, 2.03)
1911-1916	1.44(1.34, 1.55)	1.42(1.33, 1.51)	1.47(1.35, 1.59)	1.45(1.3, 1.62)	1.43(1.3, 1.58)	1.46(1.29, 1.66)
1916-1921	1.34(1.27, 1.4)	1.31(1.25, 1.37)	1.36(1.29, 1.44)	1.35(1.25, 1.46)	1.33(1.24, 1.43)	1.37(1.26, 1.49)
1921-1926	1.24(1.2, 1.3)	1.22(1.18, 1.27)	1.27(1.21, 1.32)	1.27(1.2, 1.35)	1.26(1.19, 1.33)	1.29(1.21, 1.37)
1926-1931	1.17(1.13, 1.21)	1.15(1.11, 1.19)	1.19(1.15, 1.24)	1.21(1.14, 1.27)	1.19(1.13, 1.26)	1.23(1.16, 1.29)
1931-1936	1.11(1.08, 1.15)	1.1(1.06, 1.13)	1.14(1.1, 1.18)	1.15(1.1, 1.21)	1.14(1.09, 1.2)	1.17(1.12, 1.23)
1936-1941	1.07(1.03, 1.1)	1.05(1.02, 1.08)	1.09(1.06, 1.12)	1.1(1.06, 1.16)	1.1(1.05, 1.14)	1.12(1.07, 1.18)
1941-1946	1.03(1, 1.06)	1.02(0.99, 1.04)	1.04(1.02, 1.07)	1.06(1.02, 1.11)	1.05(1.01, 1.1)	1.08(1.03, 1.13)
1946-1951	1.01(0.98, 1.04)	1(0.97, 1.03)	1.02(0.99, 1.05)	1.04(0.99, 1.08)	1.03(0.99, 1.07)	1.05(1, 1.09)
1951-1956	1(0.97, 1.03)	0.99(0.97, 1.02)	1.01(0.98, 1.03)	1.01(0.98, 1.06)	1.01(0.97, 1.05)	1.02(0.98, 1.06)
1956-1961[&]	1(1, 1)	1(1, 1)	1(1, 1)	1(1, 1)	1(1, 1)	1(1, 1)
1961-1966	1.01(0.98, 1.04)	1.02(0.99, 1.05)	1(0.97, 1.03)	0.99(0.95, 1.04)	1(0.96, 1.04)	0.98(0.94, 1.03)
1966-1971	1.02(0.99, 1.05)	1.03(1, 1.07)	1(0.97, 1.03)	0.99(0.94, 1.04)	1(0.96, 1.05)	0.97(0.93, 1.02)
1971-1976	1.03(0.99, 1.07)	1.05(1.01, 1.09)	1(0.97, 1.04)	0.99(0.94, 1.05)	1.01(0.95, 1.07)	0.97(0.91, 1.03)
1976-1981	1.04(1, 1.09)	1.06(1.01, 1.1)	1.02(0.97, 1.06)	1(0.93, 1.07)	1.01(0.95, 1.08)	0.98(0.91, 1.05)
1981-1986	1.06(1.01, 1.11)	1.08(1.03, 1.13)	1.03(0.98, 1.08)	1(0.93, 1.08)	1.02(0.94, 1.1)	0.99(0.91, 1.06)
1986-1991	1.08(1.03, 1.13)	1.1(1.05, 1.16)	1.05(1, 1.11)	1.02(0.94, 1.1)	1.03(0.96, 1.12)	1(0.93, 1.09)
1991-1996	1.11(1.06, 1.17)	1.14(1.08, 1.2)	1.09(1.03, 1.14)	1.05(0.96, 1.14)	1.06(0.97, 1.15)	1.03(0.95, 1.13)
1996-2001	1.13(1.07, 1.19)	1.15(1.09, 1.22)	1.1(1.04, 1.16)	1.06(0.97, 1.17)	1.08(0.98, 1.18)	1.05(0.96, 1.16)
2001-2006	1.15(1.08, 1.22)	1.18(1.11, 1.26)	1.12(1.06, 1.19)	1.1(0.99, 1.22)	1.11(1, 1.23)	1.08(0.98, 1.2)
2006-2011	1.19(1.11, 1.27)	1.22(1.14, 1.31)	1.15(1.08, 1.23)	1.14(1.02, 1.28)	1.16(1.03, 1.3)	1.13(1.01, 1.26)
2011-2016	1.21(1.12, 1.31)	1.24(1.14, 1.34)	1.18(1.09, 1.27)	1.18(1.03, 1.35)	1.19(1.04, 1.37)	1.17(1.02, 1.34)
2016-2021	1.25(1.12, 1.41)	1.28(1.13, 1.45)	1.23(1.09, 1.37)	1.25(1.02, 1.55)	1.26(1.01, 1.56)	1.25(1.01, 1.53)

DALYs: disability adjusted life years; CI: confidence interval.

&: reference cohort.

Supplementary Table 6. Period effect of prevelance and DALYs

Period	Relative risk of prevelance (95% CI)			Relative risk of DALYs (95% CI)		
	**Both sexes**	**Female**	**Male**	**Both sexes**	**Female**	**Male**
1992-1997	0.94(0.92, 0.96)	0.93(0.91, 0.95)	0.95(0.93, 0.97)	0.92(0.89, 0.95)	0.91(0.88, 0.94)	0.93(0.89, 0.96)
1997-2002	1.01(0.99, 1.03)	1.01(0.99, 1.03)	1.01(0.99, 1.03)	1.01(0.98, 1.04)	1.01(0.98, 1.04)	1.01(0.98, 1.04)
2002-2007[&]	1(1, 1)	1(1, 1)	1(1, 1)	1(1, 1)	1(1, 1)	1(1, 1)
2007-2012	0.96(0.94, 0.98)	0.96(0.94, 0.98)	0.96(0.94, 0.98)	0.95(0.93, 0.98)	0.95(0.93, 0.98)	0.95(0.93, 0.98)
2012-2017	0.93(0.91, 0.94)	0.93(0.91, 0.94)	0.93(0.91, 0.95)	0.91(0.89, 0.94)	0.91(0.89, 0.94)	0.92(0.89, 0.95)
2017-2022	0.93(0.91, 0.95)	0.94(0.92, 0.96)	0.93(0.9, 0.95)	0.88(0.86, 0.91)	0.89(0.86, 0.91)	0.88(0.85, 0.92)

DALYs: disability adjusted life years; CI: confidence interval.

&: reference period.

Supplementary Table 7. Estimated predicted value of ASPR and ASDALYR (2022-2031)

Year	ASPR per 100,000 (95% CI)			ASDALYR per 100,000 (95% CI)		
	Both sexes	Female	Male	Both sexes	Female	Male
2022	1304.83 (1289.31 ,1320.36)	1496.28 (1463.17 ,1529.39)	1302.72 (1271.11 ,1334.33)	58.2 (57.23 ,59.17)	63.28 (62.45 ,64.11)	55.31 (54.03 ,56.58)
2023	1087.45 (1030.16 ,1144.74)	1424.97 (1354.17 ,1495.76)	1263.83 (1201.13 ,1326.54)	48.69 (45.26 ,52.12)	54.56 (51.61 ,57.51)	48.27 (44.64 ,51.89)
2024	847.47 (722.26 ,972.68)	1361.49 (1247.92 ,1475.07)	1236.09 (1141.93 ,1330.25)	39.19 (32.03 ,46.35)	46.24 (40.04 ,52.43)	40.26 (33.17 ,47.35)
2025	617.29 (402.27 ,832.32)	1305 (1145.39 ,1464.61)	1216.3 (1091.62 ,1340.98)	31.17 (19.44 ,42.91)	39.67 (29.47 ,49.87)	31.6 (20.01 ,43.19)
2026	425.37 (106.09 ,744.64)	1254.72 (1047 ,1462.44)	1202.18 (1048.47 ,1355.89)	25.58 (8.94 ,42.22)	35.68 (21.21 ,50.16)	22.5 (5.45 ,39.56)
2027	292.44 (0 ,721.38)	1209.97 (952.92 ,1467.02)	1192.11 (1011.02 ,1373.2)	22.73 (1.34 ,44.11)	34.49 (15.94 ,53.05)	13.11 (0 ,36.51)
2028	229.27 (0 ,764.33)	1170.13 (863.12 ,1477.15)	1184.93 (978.1 ,1391.75)	22.4 (0 ,48.01)	35.76 (13.67 ,57.85)	3.53 (0 ,34.08)
2029	235.86 (0 ,866)	1134.68 (777.5 ,1491.86)	1179.8 (948.8 ,1410.8)	23.97 (0 ,53.08)	38.7 (13.82 ,63.58)	0 (0 ,32.26) *
2030	302.37 (0 ,1011.48)	1103.13 (695.91 ,1510.34)	1176.14 (922.4 ,1429.89)	26.6 (0 ,58.44)	42.34 (15.44 ,69.24)	0 (0 ,31.05) *
2031	411.23 (0 ,1181.05)	1075.04 (618.15 ,1531.93)	1173.54 (898.34 ,1448.73)	29.43 (0 ,63.29)	45.69 (17.43 ,73.96)	0 (0 ,30.43) *

ASDALYR: age-standardized disability adjusted life years rate. ASPR: age-standardized prevalence rate.

*When the predicted value for a given year is below 0, 0 is set.

Supplementary Table 8. Estimated predicted value of CPR and CDALYR (2022-2031)

Year	CPR per 100,000 (95% CI)			CDALYR per 100,000 (95% CI)		
	Both sexes	Female	Male	Both sexes	Female	Male
2022	1732.43 (1706.19 ,1758.66)	2018.43 (2001.28 ,2035.57)	1697.31 (1658.69 ,1735.93)	81.17 (79.50 ,82.84)	88.28 (86.89 ,89.66)	74.39 (72.73 ,76.04)
2023	1523.78 (1437.46 ,1610.09)	1935.34 (1875.77 ,1994.9)	1714.79 (1660.17 ,1769.41)	76.73 (72.23 ,81.24)	76.4 (71.61 ,81.19)	72.49 (69.09 ,75.89)
2024	1324.48 (1150.61 ,1498.35)	1909.91 (1793.13 ,2026.68)	1732.27 (1665.37 ,1799.16)	73.4 (65.83 ,80.97)	65.26 (55.42 ,75.09)	70.99 (65.74 ,76.24)
2025	1179.73 (903.53 ,1455.93)	1950.9 (1776.45 ,2125.35)	1749.75 (1672.5 ,1826.99)	70.9 (60.24 ,81.55)	57.23 (41.42 ,73.03)	69.8 (62.68 ,76.93)
2026	1115.92 (737.23 ,1494.62)	2039.43 (1818.76 ,2260.10)	1767.23 (1680.86 ,1853.59)	69.02 (55.35 ,82.69)	53.59 (31.72 ,75.47)	68.87 (59.89 ,77.84)
2027	1137.02 (668.07 ,1605.97)	2140.66 (1890.21 ,2391.11)	1784.7 (1690.10 ,1879.31)	67.6 (51.03 ,84.17)	54.45 (27.12 ,81.79)	68.13 (57.34 ,78.91)
2028	1226.67 (687.20 ,1766.15)	2219.99 (1954.02 ,2485.95)	1802.18 (1700 ,1904.37)	66.54 (47.20 ,85.88)	58.87 (27.13 ,90.61)	67.54 (55.01 ,80.07)
2029	1354.74 (766.16 ,1943.32)	2256.76 (1983.74 ,2529.78)	1819.66 (1710.42 ,1928.9)	65.74 (43.77 ,87.71)	65.28 (30.31 ,100.25)	67.08 (52.87 ,81.28)
2030	1486.18 (866.83 ,2105.54)	2250.3 (1973.48 ,2527.13)	1837.14 (1721.27 ,1953.01)	65.14 (40.68 ,89.61)	71.92 (34.78 ,109.05)	66.71 (50.9 ,82.53)
2031	1589.92 (952.55 ,2227.29)	2217.2 (1936.52 ,2497.87)	1854.62 (1732.48 ,1976.76)	64.69 (37.86 ,91.53)	77.26 (38.73 ,115.79)	66.42 (49.07 ,83.78)

CDALYR: crude disability adjusted life years rate. CPR: crude prevalence rate.

*When the predicted value for a given year is below 0, 0 is set.

Supplementary Table 9. Age-specific rate of prevelance and DALYs (2031)

Age	prevelance rate (95% CI)		DALYs rate (95% CI)	
(years)	Female	Male	Female	Male
0-4	391.22 (221.41 ,561.02)	491.72 (421.69 ,561.76)	14.88 (12.07 ,17.69)	13.86 (10.91 ,16.82)
5-9	882.54 (813.42 ,951.66)	809.18 (748.79 ,869.58)	33.72 (31.58 ,35.86)	29.34 (25.01 ,33.67)
10-14	555.5 (155.1 ,955.91)	928.63 (839.28 ,1017.97)	37.96 (34.07 ,41.84)	33.55 (29.89 ,37.2)
15-19	499.42 (129.27 ,869.56)	782.18 (696.22 ,868.14)	36.84 (34.68 ,39.01)	32.76 (30.82 ,34.69)
20-24	444.69 (189.45 ,699.93)	577.75 (506.3 ,649.2)	29.04 (25.52 ,32.56)	25.69 (22.58 ,28.8)
25-29	429.22 (240.89 ,617.55)	471.65 (369.09 ,574.22)	24.49 (22.48 ,26.5)	21.49 (19.58 ,23.4)
30-34	452.7 (283.51 ,621.89)	498.63 (465.53 ,531.73)	25.31 (23.42 ,27.2)	21.85 (19.95 ,23.74)
35-39	733.14 (643.82 ,822.46)	560.89 (516.58 ,605.2)	31.09 (28.84 ,33.34)	26.1 (23.95 ,28.25)
40-44	1002.51 (852.02 ,1152.99)	785.01 (725.7 ,844.31)	43.72 (40.64 ,46.79)	36.67 (34.63 ,38.71)
45-49	1539.28 (1255.47 ,1823.09)	1194.63 (937.07 ,1452.19)	74.45 (57.15 ,91.75)	62.09 (56.45 ,67.72)
50-54	2232.27 (1755.4 ,2709.14)	1944.55 (1428.86 ,2460.23)	111.74 (95.95 ,127.52)	95.27 (81.7 ,108.84)
55-59	3395.32 (2918.95 ,3871.68)	2875.54 (2509.56 ,3241.53)	163.87 (139.44 ,188.3)	137.93 (116.37 ,159.49)
60-64	3728.66 (2660.69 ,4796.64)	3537.67 (3096.07 ,3979.28)	208.71 (137.61 ,279.82)	174.62 (114.76 ,234.49)
65-69	4580.42 (2989.81 ,6171.02)	4237.66 (3285.5 ,5189.82)	280.93 (181.72 ,380.13)	235.82 (150.97 ,320.68)
70-74	5086 (3521.08 ,6650.92)	4792.62 (3527.52 ,6057.72)	342.49 (216.52 ,468.45)	290.56 (181.61 ,399.52)
75-79	6747.83 (5234.56 ,8261.1)	2635.09 (-545.03 ,5815.21)	384.48 (233.17 ,535.79)	327.9 (196.53 ,459.28)
80-84	5547.24 (3135.5 ,7958.97)	3116.85 (549.86 ,5683.83)	391.2 (231.58 ,550.82)	338.44 (201.98 ,474.9)
85-89	6544.97 (5049.52 ,8040.43)	4021.59 (1903.51 ,6139.68)	313.79 (254.38 ,373.2)	286.91 (235.89 ,337.93)
90-94	5308.39 (3871.76 ,6745.03)	5178.66 (4305.23 ,6052.1)	308.85 (186.4 ,431.29)	282.17 (190.73 ,373.61)
95+	5451.03 (4535.09 ,6366.97)	4418.38 (3859.37 ,4977.39)	271.8 (166.25 ,377.35)	249.17 (169.41 ,328.93)

DALYs: disability adjusted life years; CI: confidence interval.

Supplementary Table 10. Changes of age-specific rate for prevelance and DALYs from 2021 to 2031

Age (years)	Change of prevelance rate (%)		Change of DALYs rate (%)	
	Female	Male	Female	Male
0-4	-15	14.69	-6.25	-6.67
5-9	0	0	6.25	0
10-14	-43.21	4.5	5.56	3.03
15-19	-42.18	0	12.12	6.45
20-24	-34.37	-6.17	3.57	0
25-29	-26.42	-9.58	-4	-8.7
30-34	-21.22	-0.2	0	-4.35
35-39	6.85	-1.41	3.33	-3.7
40-44	6.25	2.21	4.76	2.78
45-49	2.19	-4.17	10.45	10.71
50-54	-0.89	3.46	10.89	13.1
55-59	7.13	7.31	13.89	14.05
60-64	-10.64	0	7.73	7.36
65-69	-15.45	-6.45	8.49	10.28
70-74	-20.91	-11.14	8.23	11.07
75-79	-4.03	-55.94	8.78	10.44
80-84	-22.48	-49.73	8.91	9.03
85-89	-4.65	-34.93	-7.92	-6.82
90-94	-14.73	-6.87	1.98	2.55
95+	-4.5	-12.45	0.74	1.22

DALYs: disability adjusted life years.

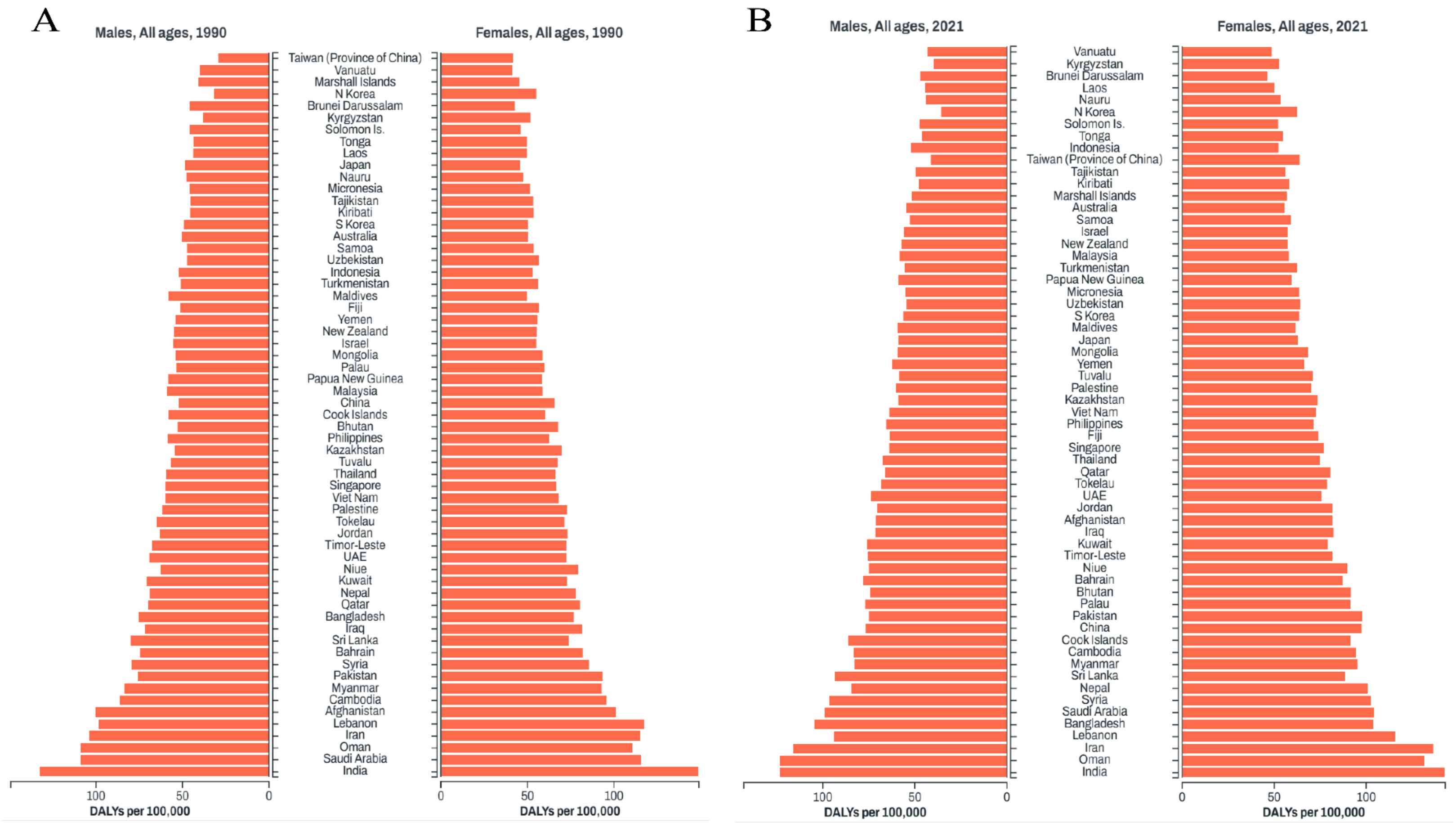

Supplementary Figure 1. DALYs rate of refraction disorders in 61 counties and regions across Asia:(A) DALYs by sexes in 1990; (B) DALYs by sexes in 2021.

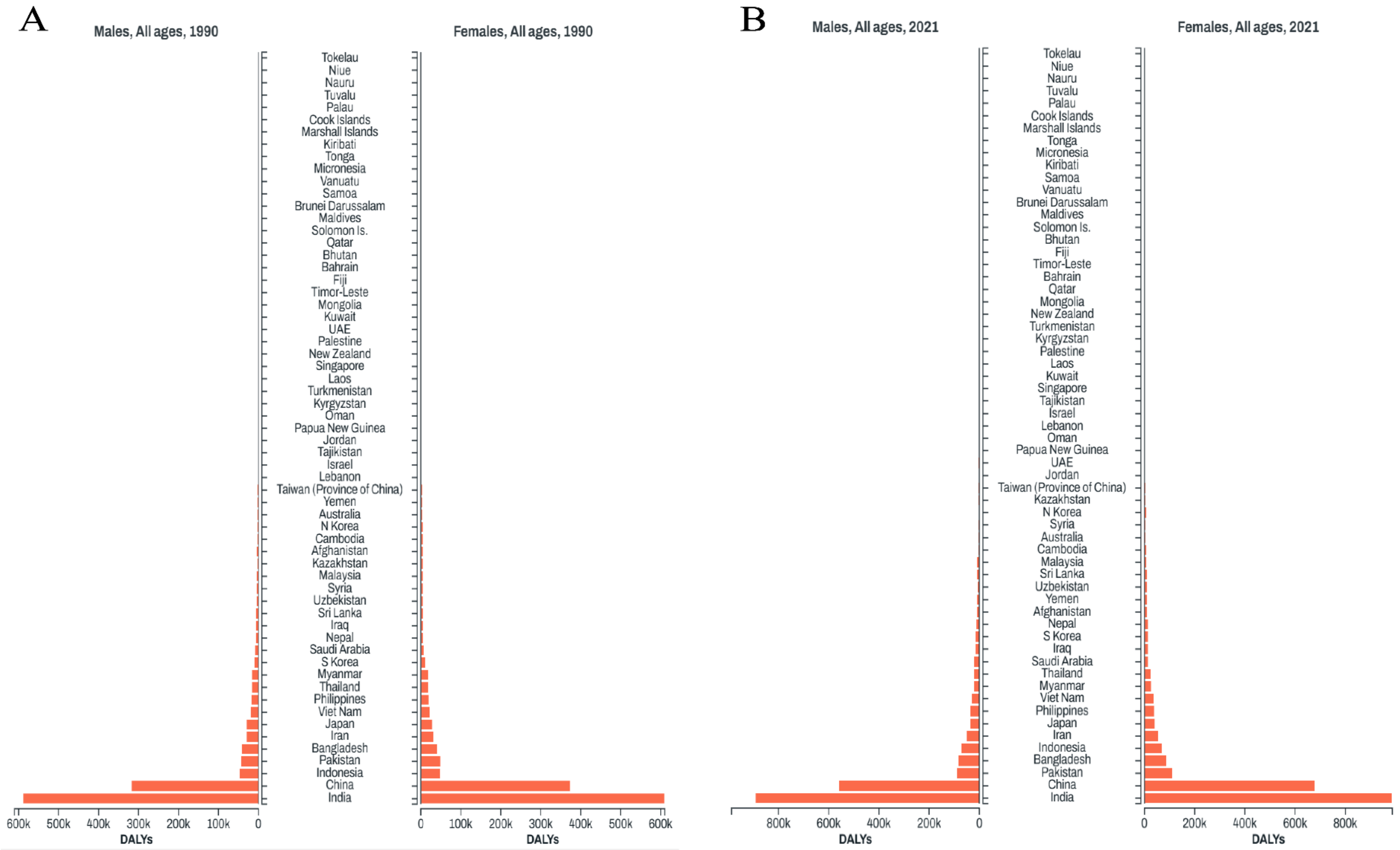

Supplementary Figure 2. DALYs number of refraction disorders in 61 counties and regions across Asia:(A) DALYs by sexes in 1990; (B) DALYs by sexes in 2021.

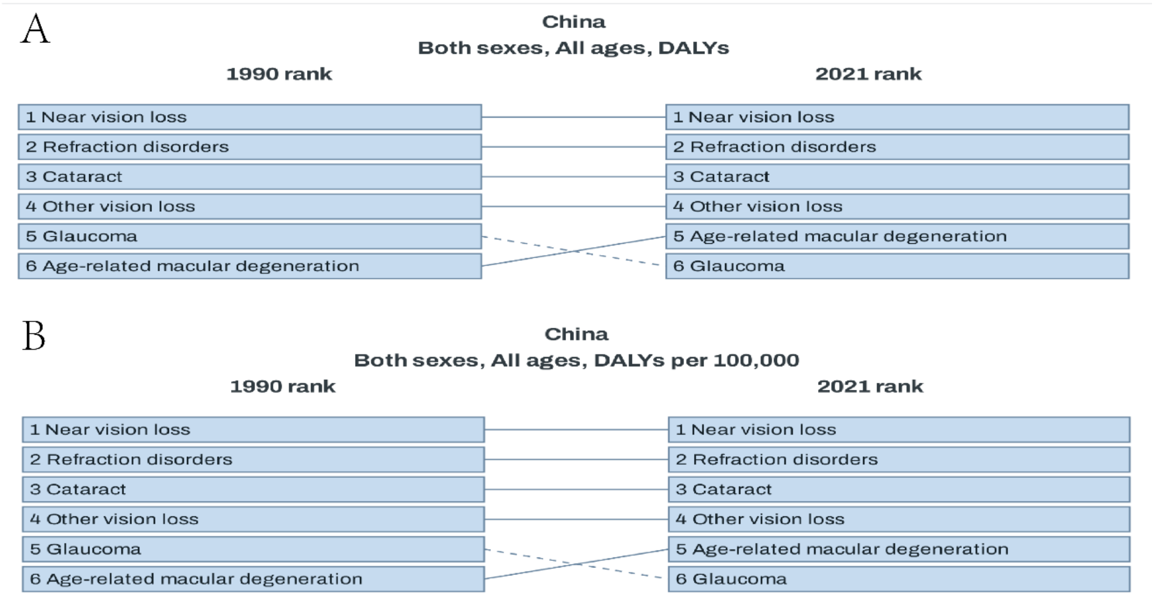

Supplementary Figure 3. The ranking changes of DALYs of refraction disorders in 6 subcategories of blindness and vision loss in China from 1990 to 2021: (A) DALYs number; (B) DALYs rate.
